# Endothelial Dysfunction in a Patient With Post-COVID-19 Syndrome and Mutation of the MTHFR Gene and Prothrombin II

**DOI:** 10.7759/cureus.92056

**Published:** 2025-09-11

**Authors:** David Martinez Juarez, Omar Gomez-Monterrosas, Francisco Zamora Rosales

**Affiliations:** 1 Radiology/Cardiovascular Imaging, Christus Muguerza Hospital Betania, Puebla, MEX; 2 Radiology/Cardiovascular Imaging, Instituto de Seguridad y Servicios Sociales de los Trabajadores al Servicio de los Poderes del Estado de Puebla (ISSSTEP), Puebla, MEX; 3 Cardiology, Angeles Hospital Puebla, Puebla, MEX; 4 Research, Christus Muguerza Hospital Betania, Puebla, MEX

**Keywords:** cardiac magnetic resonance imaging (cmri), coronary microvascular dysfunction (cmd), endothelial dysfunction, genetic thrombophilia, microvascular angina, mthfr gene, post-covid-19 syndrome, prothrombin factor ii c97g>a mutation, sars-cov-2, sticky platelet syndrome

## Abstract

Post-COVID-19 syndrome (also known as long COVID) is defined as the persistence of cardiovascular symptoms (chest pain, fatigue, palpitations) 12 weeks after the acute phase of SARS-CoV-2 infection. SARS-CoV-2 has been shown to directly infect endothelial cells through ACE2 receptors, leading to endotheliitis and vascular inflammation. In susceptible individuals, this endothelial damage may persist after viral clearance, contributing to coronary microvascular dysfunction.

Genetic predispositions (primary thrombophilias) may further aggravate endothelial injury. The prothrombin factor II G20210A mutation has been associated with an increased risk of venous and arterial thrombotic events. The MTHFR gene mutation, particularly the homozygous C677T polymorphism, is associated with reduced activity of methylenetetrahydrofolate reductase, impairing the conversion of homocysteine to methionine. This leads to hyperhomocysteinemia, which contributes to endothelial dysfunction (ED).

We report the case of a 25-year-old Mexican woman with a four-month history of chest pain and dyspnea (following a previous COVID-19 infection associated with vaccination). She presented with acute chest pain and dyspnea requiring hospitalization. Echocardiography revealed abnormal regional longitudinal strain in the basal anteroseptal wall (-7%) and basal-mid anterolateral wall (-14% and -16%, respectively), with reduced myocardial work in basal and mid-segments (379-1715 mmHg%).

Cardiac magnetic resonance imaging (CMRI) demonstrated myocardial involvement; however, coronary CT angiography (CCTA) excluded obstructive disease. Anti-SARS-CoV-2 IgG levels were markedly elevated (2893 AU/mL). Hematologic evaluation revealed abnormal platelet aggregation with platelet hyperreactivity and the identification of homozygous MTHFR C677T and heterozygous prothrombin factor II G20210A mutations.

The patient initially received prophylactic anticoagulation with apixaban during hospitalization, which was discontinued after discharge. Long-term treatment included low-dose aspirin, folic acid, B-complex vitamins, bisoprolol, and trimetazidine for angina control. At a one-year follow-up, the patient showed clinical improvement, with a reduction in the frequency and intensity of angina.

This case suggests that patients with platelet hyperreactivity, MTHFR (C677T), and prothrombin factor II (G20210A) mutations may be predisposed to developing ED and coronary microcirculatory impairment following SARS-CoV-2 infection in post-COVID-19 syndrome. We address the use of prophylactic anticoagulants in selected cases such as ours, which, although not specifically recommended by current guidelines (American Society of Hematology), may be considered after an individualized risk-benefit assessment.

## Introduction

The SARS-CoV-2 virus has shown persistent effects on endothelial function, particularly in post-COVID-19 syndrome. This RNA virus binds to ACE2 receptors, which are abundantly expressed in the vascular endothelium and pulmonary alveoli, facilitating direct viral entry and triggering endothelial damage mediated both by direct cytotoxic effects and immune-mediated mechanisms. This initial insult promotes endothelial dysfunction (ED), characterized by the loss of the endothelium’s ability to modulate vascular tone, inhibit platelet aggregation, and limit systemic inflammatory activation. These mechanisms play a central role in the pathophysiology of post-COVID-19 syndrome, and their persistence has been linked to the evolution of prolonged cardiovascular symptoms [1].

Post-COVID syndrome, as defined by the WHO and the National Institute for Health and Care Excellence (NICE), refers to the persistence of symptoms beyond 12 weeks after acute infection, in the absence of an alternative explanation. This definition emphasizes the symptomatic burden and clinical course of affected patients [2]. In contrast, the broader concept of post-acute sequelae of COVID-19 (PASC) encompasses any clinical or subclinical sequelae persisting or emerging ≥4 weeks after infection. The 2022 American College of Cardiology (ACC) Expert Consensus Decision Pathway further refined this framework by distinguishing between PASC-cardiovascular syndrome (CVS), characterized by persistent cardiovascular symptoms without demonstrable structural or functional abnormalities, and PASC-cardiovascular disease (CVD), where objective evidence of cardiovascular involvement is present [3]. This distinction is not merely semantic but clinically relevant, as it guides the diagnostic approach and therapeutic strategy. In Mexico, a study conducted on adults found that PASC was present in 21% of individuals with a prior COVID-19 diagnosis [4].

Studies have demonstrated that ED occurs not only in large vessels, such as coronary arteries, but also in the coronary microcirculation [5]. These changes can trigger prothrombotic states in the coronary microcirculation, manifesting as ischemia in the absence of obstructive coronary artery disease [6,7].

In this context, a scenario emerges in which genetic predispositions and an inflammatory state converge to promote endothelial injury. Although various autoimmune diseases, such as systemic lupus erythematosus (SLE), antiphospholipid syndrome (APS), and rheumatoid arthritis (RA), have been described as contributors to ED, in most clinical scenarios, these entities can be excluded through appropriate immunological evaluation. Nonetheless, hormonal fluctuations, particularly those associated with estrogen variability in women of reproductive age, may also play a relevant role, as they have been shown to impact vascular tone regulation and coronary microcirculation, especially in the presentation of symptoms such as catamenial angina [8].

Genomic medicine has identified mutations in the MTHFR gene and prothrombin factor II, also known as primary thrombophilias, which represent a genetic prothrombotic predisposition that may exacerbate endothelial injury. Mutations in the MTHFR gene, particularly the homozygous C677T polymorphism, are associated with reduced activity of methylenetetrahydrofolate reductase, impairing the efficient conversion of homocysteine to methionine. This leads to a state of hyperhomocysteinemia, which has been linked to ED and persistent vascular inflammation. On the other hand, the prothrombin G20210A mutation has been associated with an increased risk of venous and arterial thrombotic events due to elevated plasma prothrombin levels. This increase enhances thrombin generation and contributes to a hypercoagulable state that, by acting on receptors such as PAR-1 and PAR-2 on endothelial cells, promotes vascular inflammation, ED, and a higher risk of thrombotic events, effects that may be further aggravated in proinflammatory contexts such as post-COVID-19 syndrome [9,10].

## Case presentation

A 25-year-old Mexican woman with a history of two pregnancies complicated by threatened miscarriage, the first resulting in preterm delivery, presented for evaluation. Both parents had a history of venous thromboembolism (deep vein thrombosis (DVT)). Six months prior, she experienced a viral infection associated with COVID-19 vaccination (ChAdOx1).

**Figure 1:**
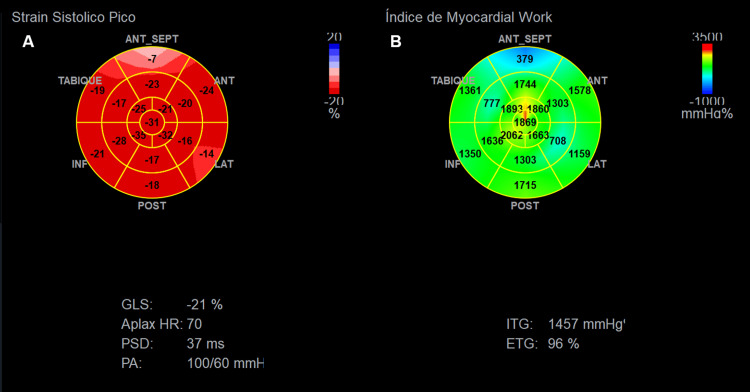
Transthoracic echocardiography (TTE) with global longitudinal strain (GLS) and myocardial work analysis Image A: Bull’s-eye plot of peak systolic strain showing segmental reduction in the basal anteroseptal wall (-7%) and basal-mid anterolateral wall (-14% and -16%, respectively; reference range: >-16%). Image B: Bull’s-eye plot of the myocardial work index demonstrating reduced regional work in basal and mid segments (379-1715 mmHg%), with a global work index of 1457 mmHg% (reference range: >1800 mmHg%). These findings suggest subclinical myocardial dysfunction.

She reported a four-month history of resting chest pain and dyspnea, which progressively worsened with emotional stress and during her menstrual cycle. Initial evaluation with an electrocardiogram (ECG) was normal, and Holter monitoring revealed no significant findings. Cardiac enzymes were within normal limits. However, transthoracic echocardiography (TTE) (Figure 1) demonstrated abnormal regional longitudinal strain in the basal anteroseptal wall (-7%) and basal-mid anterolateral wall (-14% and -16%, respectively) (ref. >-16%), with preserved global longitudinal strain of -21%. Myocardial work analysis showed reduced basal and mid-segmental values (379-1715 mmHg%) with a global work index of 1457 mmHg% (ref. >1800 mmHg%). A treadmill stress test was attempted but had to be terminated due to the exacerbation of chest pain, limiting its diagnostic value.

**Figure 2:**
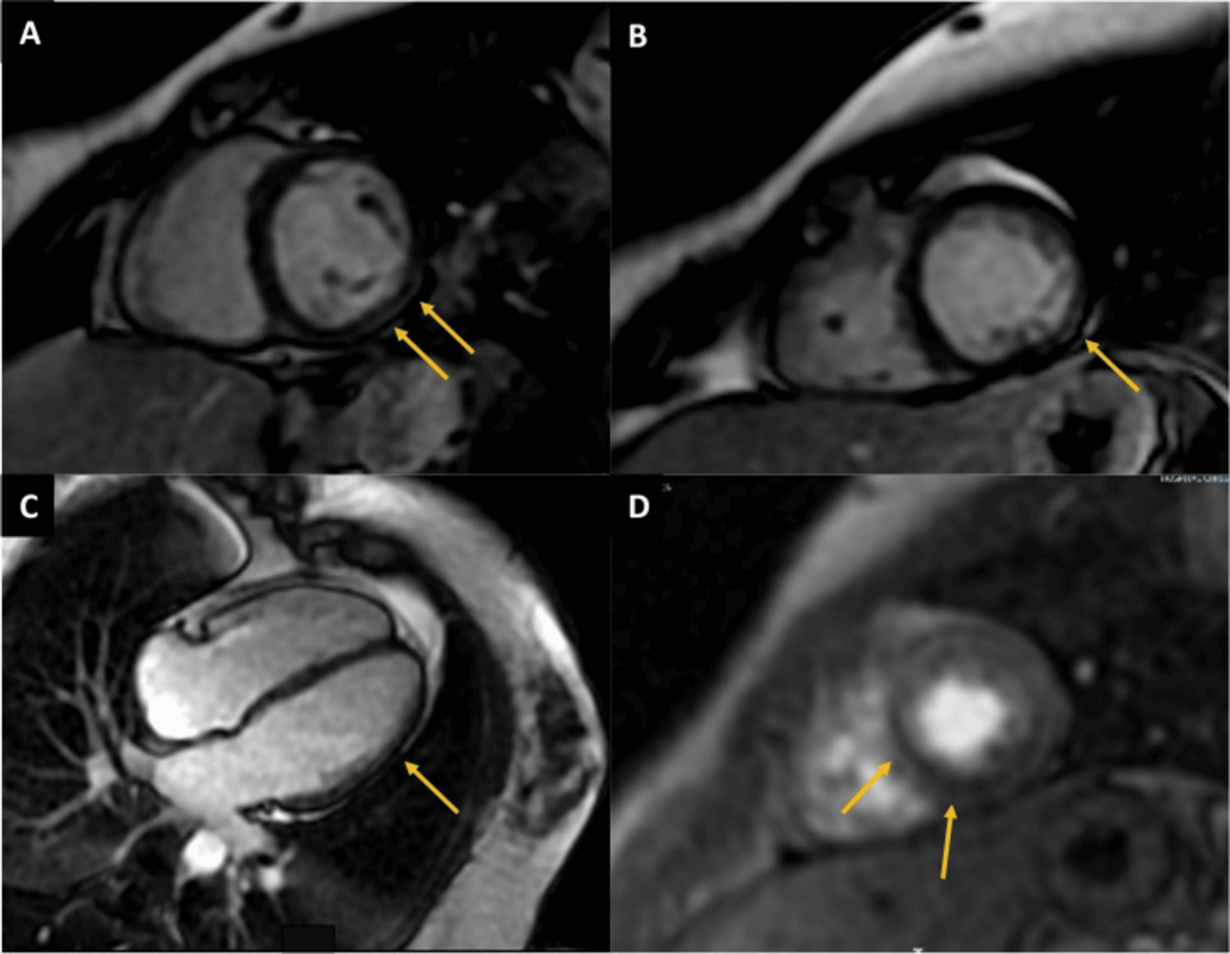
Cardiac magnetic resonance imaging performed on the patient Images A-C: Late gadolinium enhancement (LGE) sequences in short-axis (A and B) and four-chamber (C) views showing subepicardial enhancement in the posterior and inferior walls (yellow arrows), a pattern consistent with non-ischemic myocardial injury of probable inflammatory origin. Image D: First-pass perfusion sequence demonstrating a subendocardial perfusion defect in the septal and inferior walls of the mid-apical segment (yellow arrows), suggestive of ischemia without a clear coronary territory.

Cardiac magnetic resonance imaging (CMRI, Figure 2) revealed a left ventricle with normal morphology and no mobility alterations, with an ejection fraction (EF) of 66% and myocardial involvement, elevated global native T1 mapping of 1048 ms (ref. <1000 ms), global T2 mapping of 60 ms (ref. ≤55 ms), and extracellular volume (ECV) fraction of 32% (ref. <30%). Late gadolinium enhancement (LGE) demonstrated subepicardial involvement of the posterior and inferior walls, along with subendocardial perfusion defect with less than 50% transmurality in the septal and inferior walls on first-pass perfusion imaging. Based on these findings, therapy with bisoprolol, colchicine, and indomethacin was initiated.

**Figure 3:**
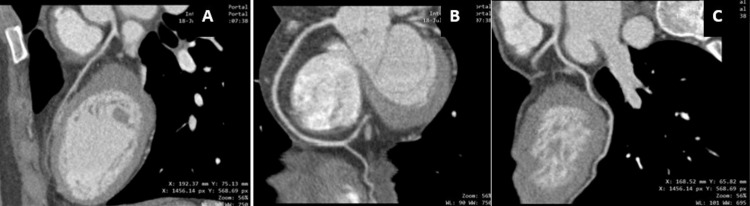
Coronary CT angiography No evidence of injury was noted. Multiplanar reconstruction image A: anterior descending artery; image B: right coronary artery; image C: circumflex artery

Several days later, the patient experienced symptom exacerbation with acute chest pain and resting dyspnea requiring hospitalization. Laboratory tests, including high-sensitivity cardiac troponin I, creatine kinase-myocardial band (CK-MB), D-dimer, ESR, CRP, and lipid profile, were within normal limits. A rapid SARS-CoV-2 test was negative, and an ECG revealed no ischemic changes. Due to her history of angina and atypical presentation, a coronary CT angiography (CCTA, Figure 3) was performed, which revealed no significant coronary stenosis.

Additional laboratory workup for SLE, APS, myositis, systemic sclerosis, Sjögren’s syndrome, and pernicious anemia excluded autoimmune causes of myocardial involvement observed on CMR.

Given persistent angina, abnormal CMR findings, and the absence of significant coronary artery stenosis in a patient without traditional cardiovascular risk factors, an immune-related platelet activation mechanism was considered. Elevated anti-SARS-CoV-2 IgG levels (2893 AU/mL) supported the suspicion of persistent immuno-platelet activation, prompting platelet aggregometry. Light transmission aggregometry demonstrated massive hyperaggregation to epinephrine and adenosine diphosphate (ADP) across all concentrations tested: epinephrine 11 μM: 10039% (ref. ≤80%); epinephrine 1.1 μM: 7315% (ref. ≤27%); ADP 1.0 μg: 998% (ref. ≤25%); ADP 0.25 μg: 210% (ref. ≤12%), consistent with platelet hyperreactivity. This finding prompted evaluation for primary thrombophilias, which revealed a homozygous C677T mutation in the MTHFR gene and a heterozygous C97G>A mutation in the prothrombin gene (factor II).

In view of the combined prothrombotic risk factors (two thrombophilic mutations, post-COVID-19 state, family history of DVT in both parents, and prior pregnancy complications with threatened miscarriage), the decision was made, albeit controversial, to initiate short-term prophylactic therapy with apixaban in combination with low-dose aspirin.

Initial treatment of angina included isosorbide; however, it was discontinued due to severe headache. Therapy was switched to calcium channel blockade (diltiazem) under the suspicion of vasospastic angina, but this yielded no significant improvement. Due to persistent symptoms and suboptimal treatment response, endothelial function was assessed by brachial artery flow-mediated dilation, which demonstrated a markedly reduced vasodilatory response of 2% (ref. >7%).

The final treatment consisted of folic acid and vitamin B complex supplementation (due to MTHFR mutation), low-dose aspirin (for sticky platelet syndrome), bisoprolol, and trimetazidine for angina control.

**Figure 4:**
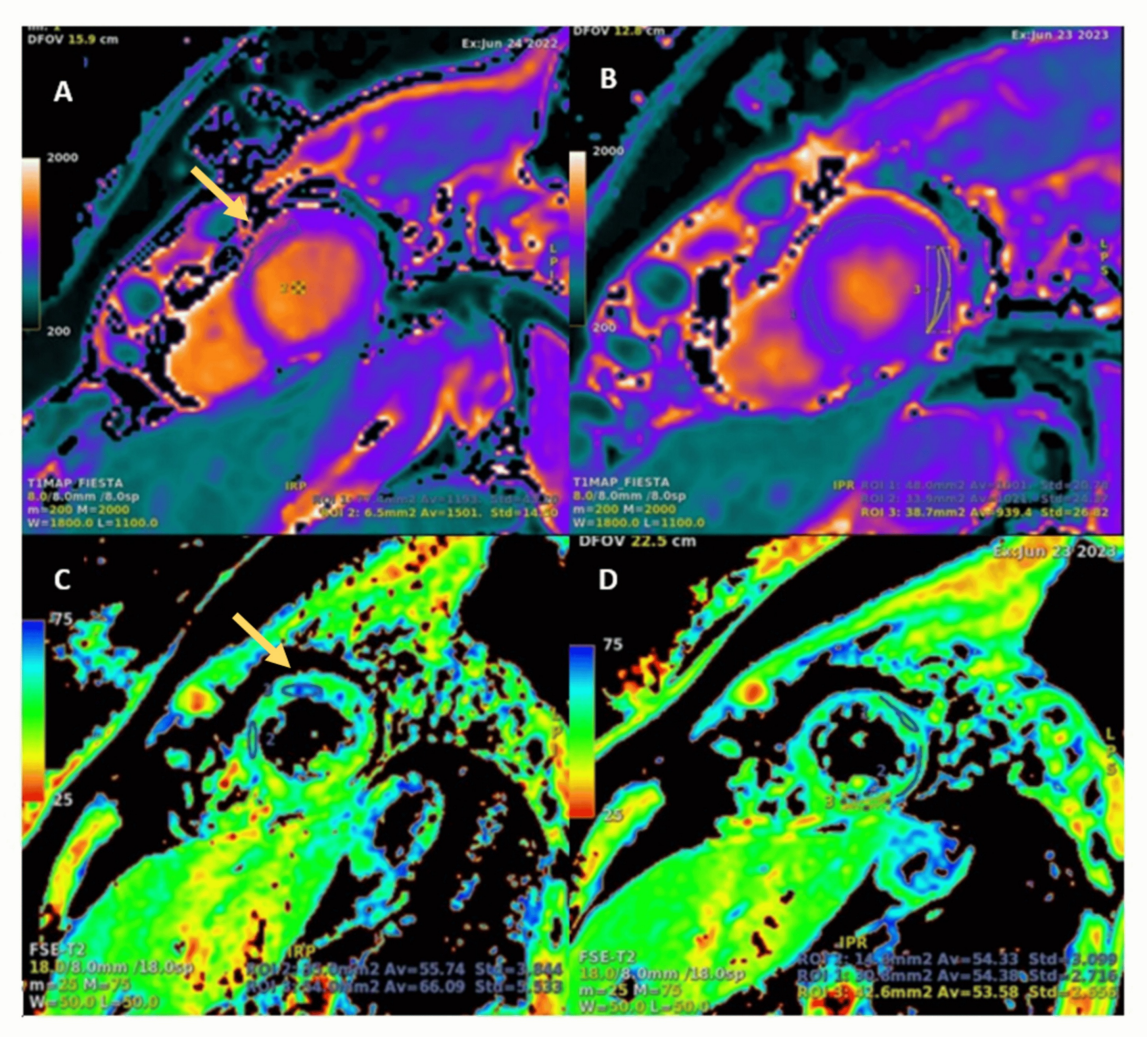
CMR T1 native and T2 mapping: baseline and follow-up study one year later Images A and C: Short-axis view of the left ventricle mid-segment. The baseline study showed increased relaxation times with focal elevation of native T1 mapping in the anterior wall (1153 ms, yellow arrows) and T2 mapping of 60 ms. Images B and D: The follow-up study performed one year later demonstrated a reduction in anterior wall relaxation times (native T1 mapping 1021 ms, T2 mapping 54 ms).

At one-year follow-up, repeat CMRI (Figure 4) demonstrated improvement with near normalization of tissue characterization: global native T1 mapping decreased from 1048 ms to 959 ms, global T2 mapping decreased from 60 ms to 52 ms, and global ECV decreased from 32% to 30%. Clinically, the patient reported a significant reduction in frequency and intensity of chest pain.

## Discussion

We present the case of a young woman with a background suggestive of a prothrombotic state and a family history of DVT. She developed angina with a four-month evolution following a respiratory infection associated with COVID-19 vaccination, consistent with post-COVID-19 syndrome. Genetic predispositions for a prothrombotic state were identified, including platelet hyperreactivity, homozygous MTHFR C677T polymorphism, and heterozygous prothrombin G20210A mutation [9,11].

In this case, we decided to use both the term "post-COVID-19 syndrome" and PASC to properly contextualize the clinical presentation. Post-COVID syndrome, according to the WHO/NICE definition [2], refers to the persistence of symptoms beyond 12 weeks with no other identifiable cause, which was consistent with the course of our patient. In contrast, the 2022 ACC consensus [3] introduced the category of PASC-CVD when objective evidence of cardiovascular involvement is present, distinguishing it from PASC-CVS, which is limited to symptoms without demonstrable abnormalities. In our patient, abnormal strain and myocardial work on echocardiography, tissue and perfusion abnormalities on CMR, and impaired endothelial function on flow-mediated dilation (FMD) supported classification as PASC-CVD. This distinction was not merely semantic but directly influenced the diagnostic approach (which included CMR and thrombophilia screening) and the therapeutic strategy, with tailored antianginal and individualized antithrombotic therapy.

Platelet hyperreactivity may be suspected in the context of COVID-19 through immune-mediated mechanisms (persistence of elevated anti-SARS-CoV-2 IgG levels) [12]. In our patient, it was demonstrated by platelet aggregometry with turbidimetry using epinephrine and ADP at low concentrations. This pattern is characteristic of the so-called sticky platelet syndrome, which has been associated with arterial and venous thrombosis. In our case, establishing this prothrombotic phenotype was important, as it converged with other factors such as the prothrombin G20210A mutation, MTHFR C677T mutation, and post-COVID-19 syndrome. The treatment of sticky platelet syndrome is based on clinical observations, with low-dose aspirin (80-100 mg) being the mainstay, as no standardized guidelines exist to date [13].

The MTHFR C677T mutation (homozygous polymorphism), although prevalent (50% to 58%) in Latin American and Asian populations [14], is not considered a clinically relevant thrombotic risk factor in the absence of hyperhomocysteinemia. However, some studies suggest that this mutation may exert a risk comparable to smoking in thrombotic disease. For instance, Jiménez et al. [15] reported that smoking and the presence of the MTHFR C677T mutation had odds ratios of 2.3 and 2.4, respectively, for the development of thrombosis.

In contrast, the prothrombin G20210A mutation is associated with an increased risk of venous and arterial thromboembolism (RR ~2-3 in heterozygotes) and with ischemic heart disease in young individuals. It also demonstrates a synergistic effect with traditional risk factors such as smoking, conferring a 43-fold increased risk of thrombotic events in smokers carrying the mutation compared with smokers without it [10].

In our patient, ED may have been influenced by genetic predisposition (thrombophilia), platelet hyperreactivity, and post-COVID-19 syndrome (sustained inflammatory response with incomplete resolution of the acute immunological phase). This is supported by the persistence of elevated anti-SARS-CoV-2 IgG levels six months after infection and vaccination. Hormonal factors may also play a theoretical role, particularly estrogen fluctuations, which modulate vascular tone and may exacerbate CMD in young women [16].

From a diagnostic perspective, imaging tools such as myocardial strain and myocardial work analysis by echocardiography are valuable for identifying early segmental or global left ventricular dysfunction, even in post-COVID-19 patients with preserved ejection fraction, thereby allowing early detection of myocardial involvement [17]. Myocardial inflammation can also be detected by CMR, even in patients with mild COVID-19 and apparent clinical recovery. Tissue characterization using native T1 mapping, T2 mapping, and ECV quantification revealed segmental elevations in our case, reflecting inflammation, edema, and interstitial expansion. Subepicardial LGE corresponded to focal areas of fibrosis. On follow-up CMR, normalization of parameters such as native T1 mapping, T2 mapping, and ECV indicated resolution of inflammation and edema, reflecting structural myocardial recovery and correlating clinically with improvement in chest pain. For example, normalization of T2 mapping relaxation times after an acute event such as reperfused myocardial infarction has been associated with edema resolution and a favorable long-term prognosis.

Although brachial artery ultrasound for EF assessment by FMD is not widely used, it may provide noninvasive evidence of ED in selected cases and serve as an early marker of CVD [18].

The 2023 American Society of Hematology (ASH) guidelines do not recommend prolonged primary anticoagulation in asymptomatic carriers of thrombophilia. However, they recognize that the prothrombin G20210A mutation carries a clinically significant risk of venous thromboembolism, particularly when additional procoagulant triggers coexist (e.g., platelet hyperreactivity and post-COVID-19 syndrome). In contrast, the MTHFR C677T mutation, although historically linked to hyperhomocysteinemia, is no longer considered a clinically relevant thrombotic risk factor in heterozygous or homozygous carriers with normal homocysteine levels [19]. Thus, it should not be used as the sole basis for antithrombotic therapy, although it may still contribute to ED. The ASH 2023 guidelines, along with recommendations from the American College of Obstetricians and Gynecologists (ACOG) [20], emphasize that MTHFR mutation status alone should not guide antithrombotic management due to its limited predictive value.

With respect to angina management, isosorbide was initially prescribed but was poorly tolerated due to severe headaches. According to the 2020 European Association of Percutaneous Cardiovascular Interventions (EAPCI) expert consensus on Ischemia with Non-Obstructive Coronary Arteries (INOCA), calcium channel blockers are recommended as first-line therapy. However, our patient did not achieve clinical improvement, leading to a therapeutic switch to a combination of bisoprolol and trimetazidine. The latter is considered a second-line antianginal agent that improves cellular tolerance to ischemia by maintaining metabolic homeostasis [21].

## Conclusions

This case suggests that patients with platelet hyperreactivity, MTHFR (C677T), and prothrombin factor II (G20210A) mutations may be predisposed to developing ED and coronary microcirculatory impairment following SARS-CoV-2 infection in post-COVID-19 syndrome. We acknowledge the limitation of reporting a single case but highlight the importance of a multidisciplinary diagnostic approach and individualized treatment in young patients with angina and no traditional cardiovascular risk factors in the post-COVID-19 setting.
